# Spiritual Healing

**Published:** 1923-10

**Authors:** 


### SPIRITUAL HEALING.

A NORWICH clergyman has been telling a congregation in Westminster Abbey that he has recently witnessed a number of wonderful cures by "spiritual healing." Sight has been restored to the blind, and an arm withered and useless for sixteen years suddenly became perfectly whole. Moreover, "we have seen cancers disappear within twenty minutes." We make bold to say at once that, whatever it was that disappeared, it was not cancer, and it is not surprising that these remarkable statements should have produced a large amount of newspaper correspondence from medical and scientific men and clergymen. The former are, naturally, sceptical of claims which appear, on the face of them, to be extravagant, and Sir Bryan Donkin has gone to the root of the matter by asking for proofs. No such proofs have yet been forthcoming, and those who are acquainted with the literature, at once copious and curious, of the subject know how often the same demand has been made in the past and how rare has been any convincing response. Faith-healing is almost as old as humanity, and it has been a happy hunting-ground for the charlatan and the imposter. This is not to say that faith and prayer are incapable of removing certain classes of disorders. There is substantial reason for believing, in given circumstances, in their efficacy, and there is abundant experience to show that the doctor and the priest may work together with results which neither of them could achieve separately. But healing by faith, whether it be by the laying on of hands, accompanied by prayer, in a church, or by the methods of Christian Science, lies, and long has lain, under suspicion. Nervous excitement and spiritual exaltation may, and undoubtedly sometimes do, produce remarkable consequences. Whether they have ever produced permanent cures is another question. Science has first to be satisfied that the disease was there to be cured, and then to be satisfied that it actually is cured.

The Church of England, in spite of much temptation, has wisely preserved a cautious attitude towards the subject. The Committee on Spiritual Healing appointed by the last Lambeth Conference has declared that, while "the Church must recognise methods of faith-healing in the treatment of bodily disease," it must yet "be careful to apply such methods in accordance with scientific discovery and analyses," and adds that "the indiscriminate or unintelligent appeal to faith may bring some immediate relief, but may do more harm than good in the end." This is sound common sense and strikes a sadly-needed note of warning, while leaving wide open the door to those psychological possibilities which were indicated years ago by Sir Thomas Clouston when he expressed his conviction that "in time, through mental influences, some diseases now regarded as incurable will be healed" since "such influences largely control nutrition, and many organic diseases result from malnutrition." Admitting, therefore, the strong possibility that certain affections may be cured by influences loosely described as "mental" or "spiritual"—they are very far from being identical—two things are clearly necessary if we are to form a just judgment. First, it must be ascertained beyond doubt that the patient actually is suffering from this or that disease, and, second, we must know, equally definitely, whether the disease has been ameliorated or cured by methods independent of the physician or the surgeon. Hitherto it has been nobody's business to certify to either of these things.

The claims that are made for healing outside science are capable of being proved or disproved, and it is time that they were put to the touch in circumstances which exclude the possibility of error. It is not for man to suggest limits to the Divine power of healing, but reverence, as well as a desire for the ascertainment of truth, demands that we should be sure that we are dealing with that power and not with some form of conscious or unconscious imposture. What the world wants to know is what happens eventually to people whose sight is said to have been instantaneously restored, or whose paralysed limbs have resumed their activity at the touch of the healing hand. What is their condition six or twelve months later ? Are they still "cured," or have they relapsed ? We hear of alleged instantaneous healings, but we rarely, if ever, hear anything of the relapse, slow or rapid, which must inevitably happen in many cases. Until these matters have been put to scientific proof the world will have to continue to listen alternately to extravagant claims on the one hand and to flat refusals to believe on the other. Vast numbers of people are sure that there is "something" in forms of healing that are outside our science, but those who think clearly are anxious to know not only exactly what that "something" is, but to be able to form a well-founded opinion of its boundaries.

